# Influence of Reduced Graphene Oxide on the Electropolymerization of 5-Amino-1-naphthol and the Interaction of 1,4-Phenylene Diisothiocyanate with the Poly(5-Amino-1-naphtol)/Reduced Graphene Oxide Composite

**DOI:** 10.3390/polym12061299

**Published:** 2020-06-05

**Authors:** Mihaela Baibarac, Monica Daescu, Marcela Socol, Cristina Bartha, Cătălin Negrila, Szilárd N. Fejer

**Affiliations:** 1 Laboratory of Optical Processes in Nanostructured Materials, National Institute of Materials Physics, Atomistilor Street 405A, P.O. Box MG-7, RO77125 Magurele, Romania; monica.daescu@infim.ro (M.D.); marcela.socol@infim.ro (M.S.); 2 Faculty of Applied Chemistry and Materials Science, University Politehnica of Bucharest, Gh. Polizu St 1-7, 011061 Bucharest, Romania; 3 Magnetism and Superconductivity Laboratory, National Institute of Materials Physics, Atomistilor Street 405A, P.O. Box MG-7, RO77125 Magurele, Romania; cristina.bartha@infim.ro; 4 Nanoscale Condensed Matter Laboratory, National Institute of Materials Physics, Atomistilor Street 405A, P.O. Box MG-7, RO77125 Magurele, Romania; catalin.negrila@infim.ro; 5 Pro-Vitam Ltd., Muncitorilor Street 16, 520032 Sfantu Gheorghe, Romania; fejersz@gmail.com

**Keywords:** poly(5-amino-1-naphthol), reduced graphene oxide, functionalization

## Abstract

A new composite base on reduced graphene oxide (RGO) and poly(5-amino-1-naphthol) (P5A1N) was synthesized by the electrochemical polymerization of 5-amino-1-naphthol (5A1N) in the presence of HClO_4_ and H_4_SiW_12_O_40_ onto the surface of Au electrode covered with the RGO sheets. The linear dependence of the current densities of the anodic and cathodic peaks with the scan rate of the potential range (0; 0.8) V vs. SCE, reported during electropolymerization of 5A1N, indicates an electron transfer that is controlled by diffusion. A covalent functionalization of the RGO sheets with P5A1N is argued by: (i) the simultaneous disappearance of the IR band at 1584 cm^−1^ and the appearance of the new IR bands at 812, 976 and 3744 cm^−1^, and (ii) the appearance of two Raman lines at 738 and 1428 cm^−1^. An application of the RGO sheets covalently functionalized with P5A1N is demonstrated to support 1,4-phenylene diisothiocyanate (PDITC), a compound used as a cross-linking agent for various biological applications. The chemical adsorption of PDITC onto the RGO sheets covalently functionalized with P5A1N, which involves the appearance of new functional groups of the type thiourea, was proven by Raman scattering and IR spectroscopy.

## 1. Introduction

The electrochemical polymerization of 5-amino-1-naphthol (5A1N) was carried out for the first time in 1991, when the macromolecular compound poly(5-amino-1-naphthol) (P5A1N) with a nanofibrillar structure having a purplish gray color was obtained [1]. The main applications developed using this macromolecular compound were related to: (i) the corrosion protection of the mild steel covered with P5A1N [2,3]; (ii) voltage-regulator devices [4]; (iii) amperometric NO microsensors [5,6] and (iv) catalysis [7]. Another application of P5A1N envisaged to be developed in this work is in the field of the composite materials based on reduced graphene oxide (RGO) sheets functionalized with macromolecular compounds. The main experimental techniques used until now for the characterization of P5A1N were infrared absorption spectroscopy [8], Raman scattering [9,10,11], UV–Vis spectroscopy [10], X-ray photoelectron spectroscopy [11] and atomic force microscopy [12]. As shown in several articles (e.g., [13,14]), IR absorption spectroscopy and Raman scattering were reported to be two tools useful in assessing of the functionalization processes of the RGO sheets with macromolecular compounds. Therefore, those two techniques were used in the present study as well. A relevant role in the P5A1N electroactivity was reported to be played by anions as those coming from camphorsulphonic acid, H_3_PMo_12_O_40_ and so on [7,15]. In this work, the positive charges generated during electrosynthesis of P5A1N will be compensated by the H_4_SiW_12_O_40_ heteropolyanions. The choice of H_4_SiW_12_O_40_ was made considering the less oxidative properties of this heteropolyacid, compared to other compounds such as H_3_PW_12_O_40_ or H_3_PMo_12_O_40_ [16], a fact that will allow the preservation of the RGO sheets’ structure after the P5A1N electrosynthesis. Taking into account this progress, the following issues are debated in this article: (i) highlighting the interaction of 5A1N with the RGO sheets by Raman scattering, IR spectroscopy, photoluminescence, X-ray photoelectron spectroscopy and thermogravimetric analysis; (ii) illustration of the influence of the RGO sheets on the electrochemical polymerization of 5A1N and the understanding of the functionalization process of the RGO sheets with P5A1N considering the Raman scattering and IR spectroscopy studies; and (iii) assessing of the adsorption of 1,4-phenylene diisothiocyanate (PDITC) onto the RGO sheets functionalized with P5A1N in order to prepare new potential platforms for the detection of cancer biomarkers.

## 2. Materials and Methods

Some of the compounds used in this work, i.e., 5-amino-1-naphthol (5A1N), H_4_SiW_12_O_40_ xH_2_O_,_ HClO_4_, graphite, dimethylformamide (DMF), C_2_H_5_OH, sodium nitrate, H_2_SO_4_, PDITC, KMnO_4_ and H_2_O_2_ were purchased from Sigma-Aldrich. 

RGO was obtained by the interaction of hydrazine with graphene oxide prepared according to D.C. Marcano’s and S. Eiger’s procedure [17,18]. 

The mechanico-chemical reaction of 5A1N with the RGO sheets was carried out in the absence of light, the two constituents being non-hydrostatically compressed at the pressure of 0.58 GPa, for 5 min, when the 5A1N/RGO platelets having 0.5, 1 and 2 wt % RGO were obtained. 

The RGO sheets functionalized with P5A1N doped with H_4_SiW_12_O_40_ heteropolyanions were prepared by electropolymerization of 5A1N onto the Au electrode covered with the RGO sheets. The cyclic voltammetry studies were carried out by the immersion of the working electrode into a solution consisting of 5A1N (10^−3^, 5 × 10^−3^ or 10^−2^ M), H_4_SiW_12_O_40_ (10^−3^, 5 × 10^−3^ or 10^−2^ M) and 0.1 M HClO_4_. The potential range used to record the cyclic voltammograms was between 0 and +750 mV vs. SCE electrode. Various sweep rates equal to 100, 50, 40, 30, 20, 10, 5 and 2 mV s^−1^ were used. The electrochemical studies were carried out using a potentiostat/galvanostat, purchased from Radiometer Analytical (Villeurbanne, France), Voltalab 80 model. 

The PDITC deposition on the RGO sheets functionalized with P5A1N doped with H_4_SiW_12_O_40_ heteropolyanions was achieved by the drop casting method using a PDITC-C_2_H_5_OH solution with the concentration of 1 mg/mL.

Raman spectra of 5A1N before and after the interaction with the RGO sheets; P5A1N doped with H_4_SiW_12_O_40_ heteropolyanions; and the RGO sheets functionalized with P5A1N doped with H_4_SiW_12_O_40_ heteropolyanions in initial state and after their interaction with PDTIC were obtained with a FTRaman spectrophotometer, from Bruker (Bruker Optik GmbH, Ettlingen, Germany).

For recording IR spectra of 5A1N before and after the interaction with the RGO sheets; P5A1N doped with H_4_SiW_12_O_40_ heteropolyanions; and the RGO sheets functionalized with P5A1N doped with H_4_SiW_12_O_40_ heteropolyanions in initial state and after their interaction with PDTIC—a FTIR spectrophotometer, from Bruker (Billerica, MA, USA), Vertex 80 model was used. 

The characterization of 5A1N and the 5A1N/RGO composite by X-ray photoelectron spectroscopy (XPS) was carried out in a SPECS spectrometer (SPECS Gmbh, Berlin, Germany) by using the Mg Kα source at 12 kV and 24 mA in a vacuum chamber at 2 × 10^−8^ mbar. The spectrometer is equipped with a PHOIBOS 150 hemispherical analyzer with an ultimate resolution of 0.44eV (measured as the FWHM of Ag3d^5/2^ line recorded in optimum conditions) and with a nine channel electron detector. The spectra were recorded using a pass energy of 50 eV for the extended spectra and 5 eV for the spectral lines. The fittings of the spectra were made in the Spectral Data Processor software using Voigt functions and specific sensitivity factors.

The thermal stabilities of the RGO sheets, 5A1N and the 5A1N/RGO composite were investigated using a SETARAM SETSYS Evolution 18 device in a thermogravimetry-differential thermal analyzer (TG-DTA, SETARAM Instrumentation, Caluire, France) mode from room temperature up to 800 °C. The samples were measured in an inert atmosphere of argon with a 16 mL/min flow gas rate and a heating rate of 10 °C/min. The accuracy of heat flow measurements was ±0.001 mW with a temperature precision of ±0.01 °C.

The atomic force microscopy (AFM) images of the Au electrode, before and after the deposition of the RGO sheets onto its surface, were collected with a Nanonics Multiview 4000 microscope (Nanonics Imaging Ltd., Jerusalem, Israel) working in tapping mode in the following conditions: 20 nm probe diameter, 33.08 kHz vibration frequency, 1540 factor of merit and 10 ms/point scan speed. The roughness parameters (root mean square—RMS; and roughness average—Ra) were calculated from the AFM scans. 

## 3. Results

### 3.1. Chemical Interaction of the RGO Sheets with 5A1N

**Figure 1:**
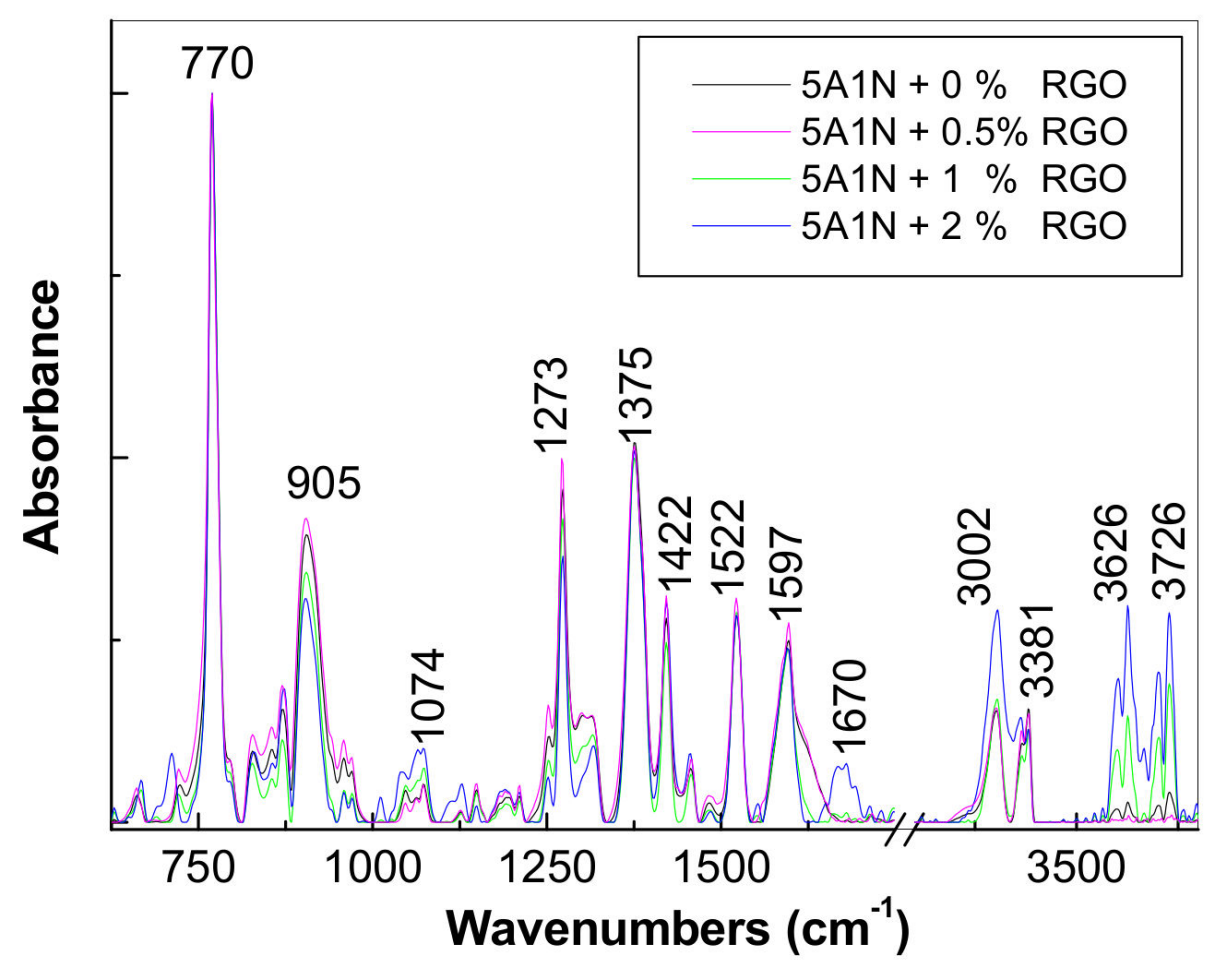
IR spectra of 5A1N (black curve) and the 5A1N/RGO mixtures with RGO sheets concentrations equal to 0.5 wt. % (magenta curve), 1 wt. % (green curve) and 5 wt. % (blue curve).

In Figure 1, one observes that the IR spectrum of 5A1N is characterized by eight bands peaking at 770, 905, 1065, 1271, 1375–1422, 1522, 1597 and 3002–3381 cm^−1^, these being assigned to the out-of-plane bending vibrations of the C–H bonds of naphthalene nuclei, the stretching vibrations of the C–O bonds, the in-plane bending vibrations of the C–H bonds of naphthalene nuclei, the stretching vibrations of the C–N bonds in secondary amine, the stretching vibrations of the C–O bonds of phenolic compounds overlapping the in-plane bending vibration of the O–H bonds, the stretching vibrations of the C=C and C–H bonds of naphthalene and the stretching vibrations of N–H bonds [1]. 

The interaction of 5A1N with the RGO sheets induces in the IR spectra shown in Figure 1, the following changes: (i) a gradual decrease in the absorbance of the IR band at 905 cm^−1^ simultaneously to the increase in the absorbance of the IR band at 3002 cm^−1^ with increasing RGO concentration in the 5A1N/RGO mixture; and (ii) the appearance of new IR bands peaking at 1670 and 3626–3726 cm^−1^, which were assigned to the benzene rings and OH bond stretching [19]. Additional information concerning the interaction of 5A1N with RGO is shown in the following by Raman spectroscopy.

**Figure 2:**
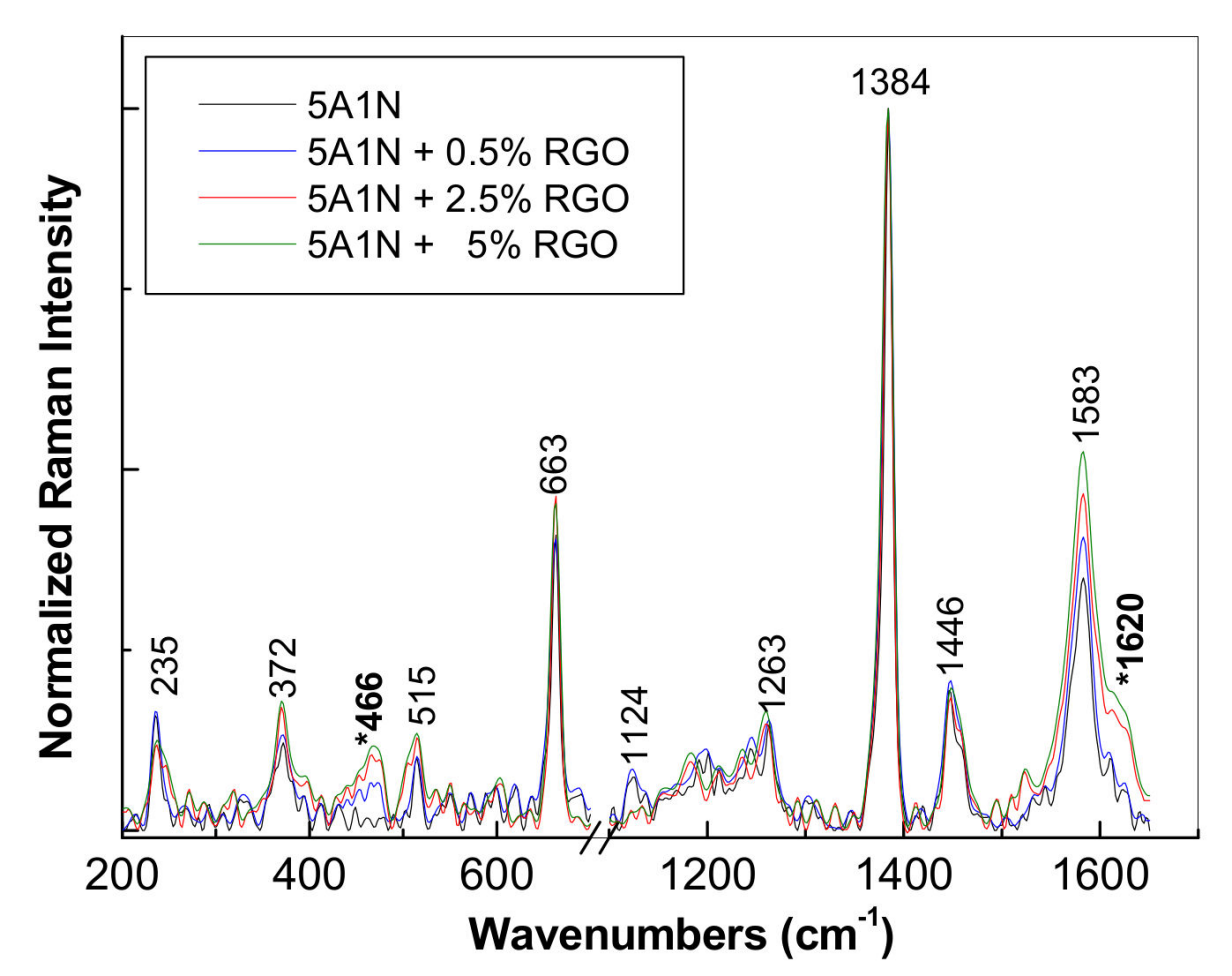
Raman spectra of 5A1N (black curve) and the 5A1N/RGO mixtures with RGO sheets concentrations equal to 0.5 wt. % (blue curve), 2.5 wt. % (red curve) and 5 wt. % (green curve).

**Scheme 1:**
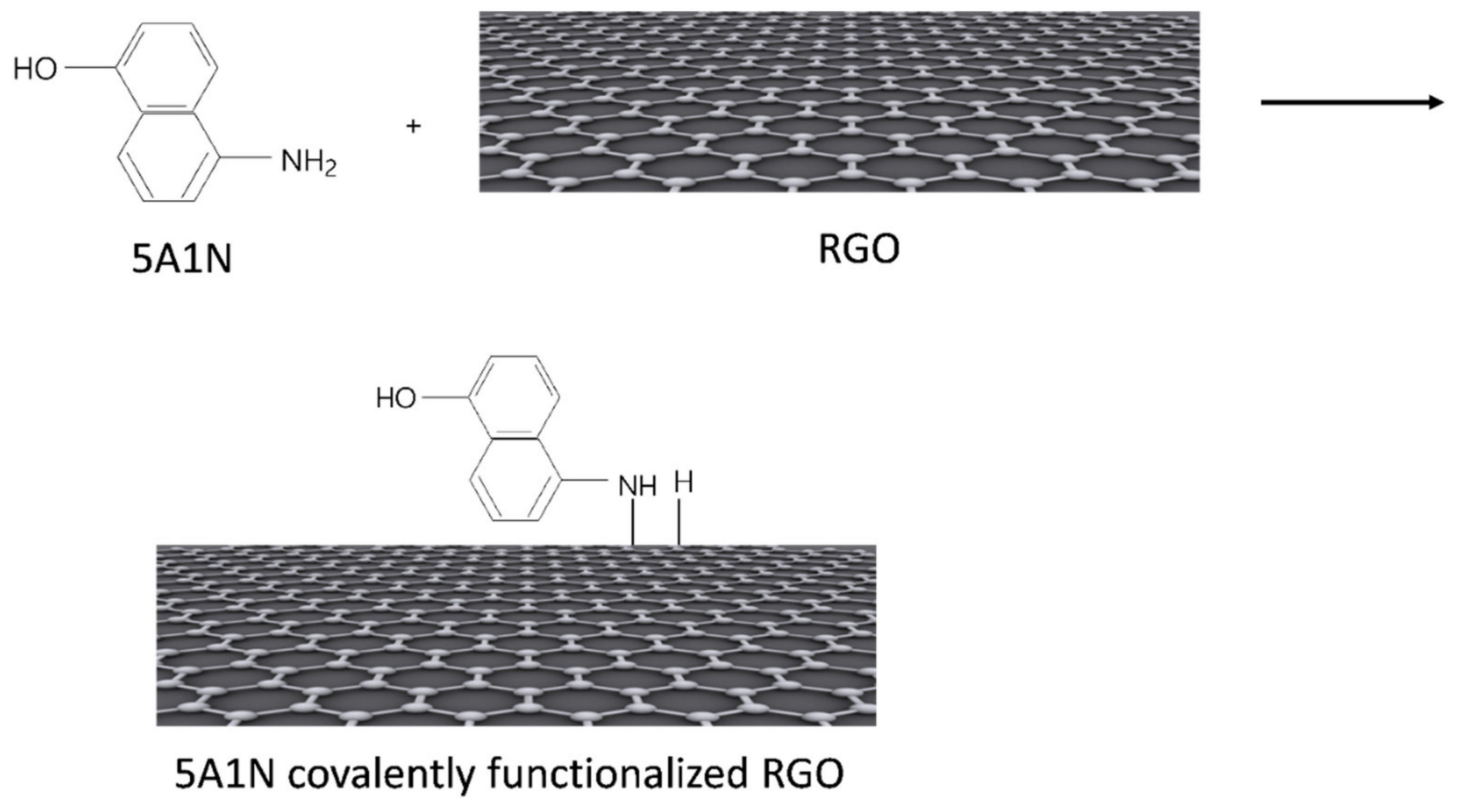
The chemical interaction of 5A1N with the RGO sheets.

According to Figure 2, the main Raman lines of 5A1N have peaks at 235–372-515, 663, 1124, 1263, 1384, 1446 and 1583 cm^−1^, are assigned to the vibrational modes of: the deformation of the C–C–C bond in naphthol, the deformation of the C–C bond in aromatic ring, the C–H bond in aromatic ring, the stretching of the C–N bond, the stretching of the C–C and C–O bonds, the stretching of the C–C bond and the bending of the C=C bonds [11]. When increasing the RGO sheets concentration in the 5A1N/RGO mixture from 0 to 5 wt. %, the following changes are remarked on from Figure 2: (i) a decrease of the ratio between the intensities of the Raman lines at 1384 and 1446 cm^−1^ from 5.24 to 3.29; (ii) an increase in the intensity of the two new Raman lines peaked at 466 and 1620 cm^−1^; and (iii) the variation in the value of the ratio between the intensities of the Raman lines peaked at 1384 and 1583 cm^−1^ from 2.86 to 2.13. The Raman lines at 466 and 1620 cm^−1^ are assigned to the vibrational modes of the deformation of the aromatic ring and the C–N–C bonds [9] which can be explained only if we accept that the interaction of 5A1N with the RGO sheets takes place according to Scheme 1. According to Scheme 1, the chemical interaction of 5A1N with the RGO sheets leads to a covalent functionalization of the RGO sheets with 5A1N.

In our opinion, Scheme 1 can also explain the appearance of the IR bands peaked at 670 and 3626–3726 cm^−1^, as the covalent functionalization of the RGO sheets with 5A1N induces significant hindrance effects of the aromatic rings and the OH bond coming from 5A1N. 

**Figure 3:**
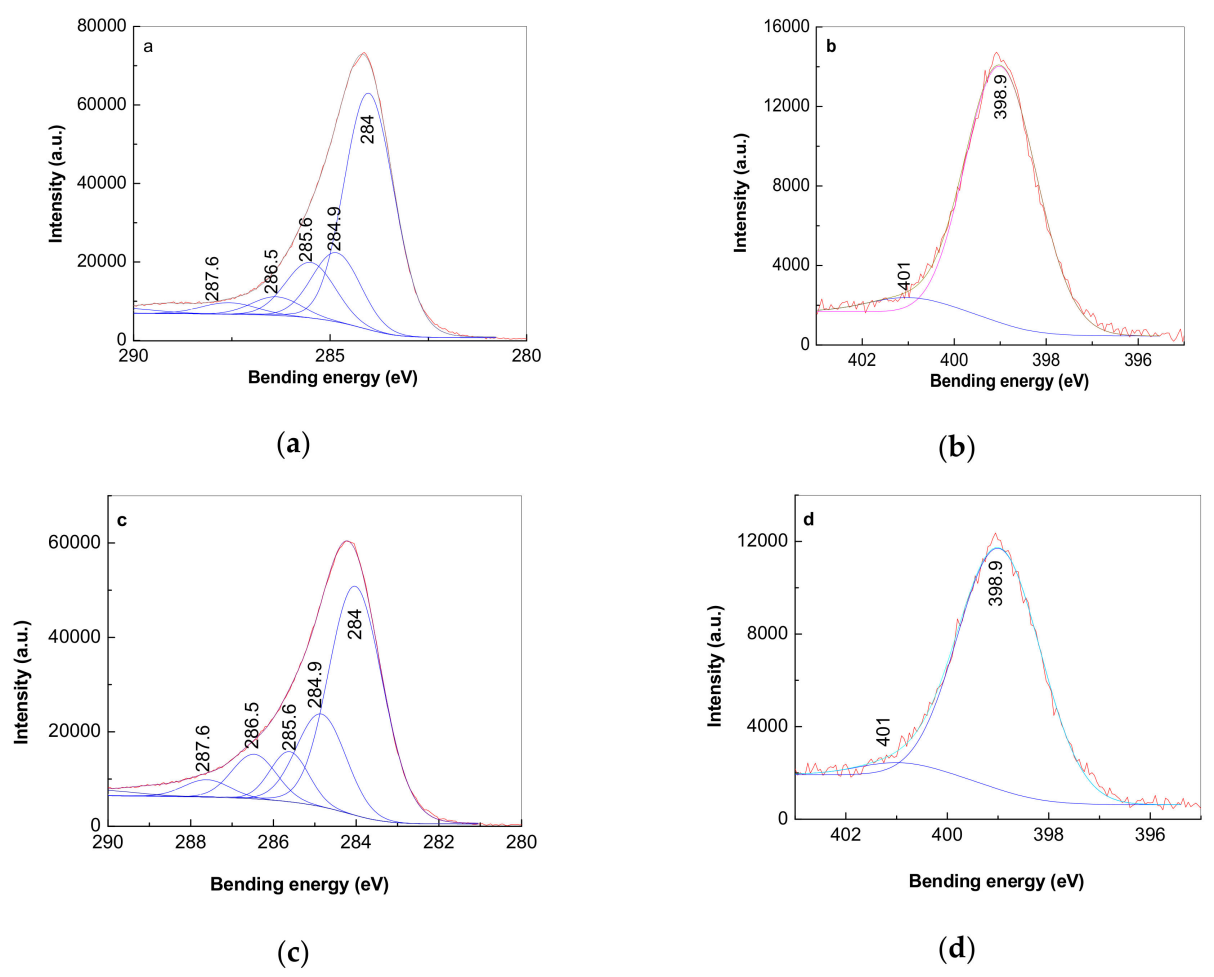
XPS C1s spectra of 5A1N (**a**) and the 5A1N/RGO composite (**c**). XPS N1s spectra of 5A1N (**b**) and the 5A1N/RGO composite (**d**).

Other supporting information for the chemical interaction of 5A1N with the RGO sheets, in the following, is shown by XPS spectroscopy and thermogravimetric analysis. Figure 3 displays C1s spectra of 5A1N and its composite with RGO. According to Figure 3a, the C1s spectrum of 5A1N shows a profile which can be deconvoluted in five bands with maxima at 284, 284.9, 285.6, 286.4 and 287.6 eV, which were assigned to sp^2^ C=C bonds, sp^3^ C–C bonds, C–N bonds, C–O bonds and C=O bonds [20]. Figure 3b shows the N1s spectrum that can be deconvoluted in two bands with the peaks at 398.9 and 401 eV, assigned to the neutral amine nitrogen atoms (R–NH_2_) and H-bonding between amine groups [21,22]. In comparison with the C1s spectrum of 5A1N, in the case of the 5A1N/RGO composite we remark a significant variation in the intensity of the bands peaked at 284 eV and 284.9 eV, which induces a change of the ratio between the intensities of the two bands from 2.82 (Figure 3a) to 2.14 (Figure 3c). A careful analysis of the N1s spectra of 5A1N (Figure 3b) and its composite with RGO (Figure 3d) indicates that the ratio between the intensities of the bands peaked at 398.9 and 401 eV varies from 5.96 (Figure 3b) to 4.84 (Figure 3d). Taking into account this last variation, the decrease in the weight of the primary amine bonds compared to the hydrogen bonds between the amine groups can be explained as a result of the transformation of the primary amine groups into secondary amine groups, as illustrated in Scheme 1.

**Figure 4:**
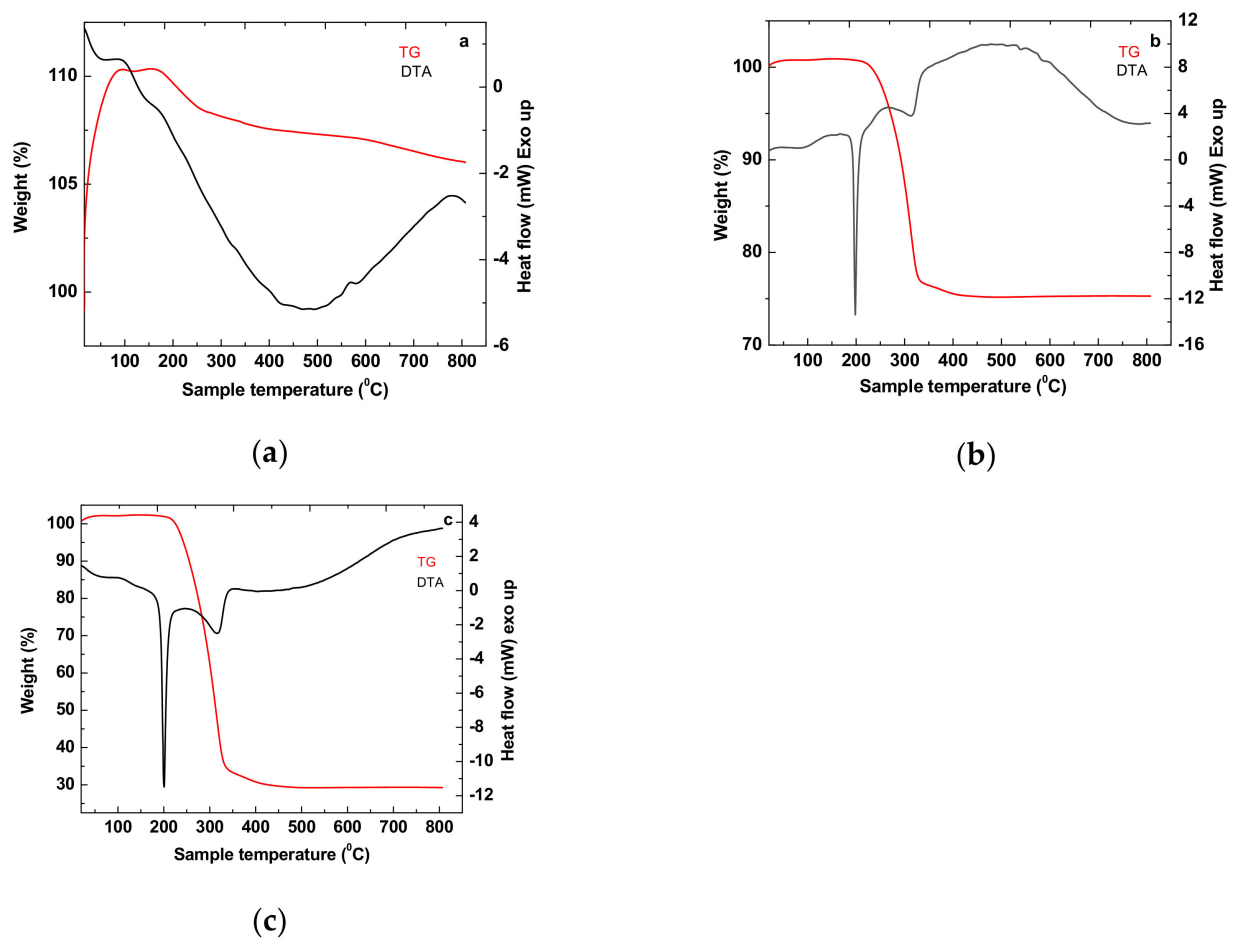
TG-DTA curves of the RGO sheets (**a**), 5A1N (**b**) and the 5A1N/RGO composite (**c**).

Concerning the thermal stability of 5A1N, the RGO sheets and the 5A1N/RGO composite, the following variations were observed: (i) Figure 4a shows thermal stability of RGO up to 800 °C. The slight increase in mass of about 8–10 wt. %, visible on the TG curve, can be attributed to a process of gas adsorption on the nanoparticles surface. (ii) Figure 4b confirms the mechanism of thermal decomposition which highlights the two endothermic maxima at 200 °C and 350 °C and a mass loss of about 25%. (iii) Figure 4c highlights according to the TG curve of the 5A1N/RGO sample a mass loss of approximately 65% due to the thermal degradation of the organic compound. This thermal behavior takes place in two stages according to the energy curve (DTA) which highlights the presence of two endothermic peaks with maxima around 200 °C and 350 °C. Above 400 °C, the 5A1N/RGO sample becomes stable in temperature.

### 3.2. The Electrochemical Polymerization of 5A1N onto the Blank Au Electrode and the Au Plate Covered with the RGO Sheets

**Figure 5:**
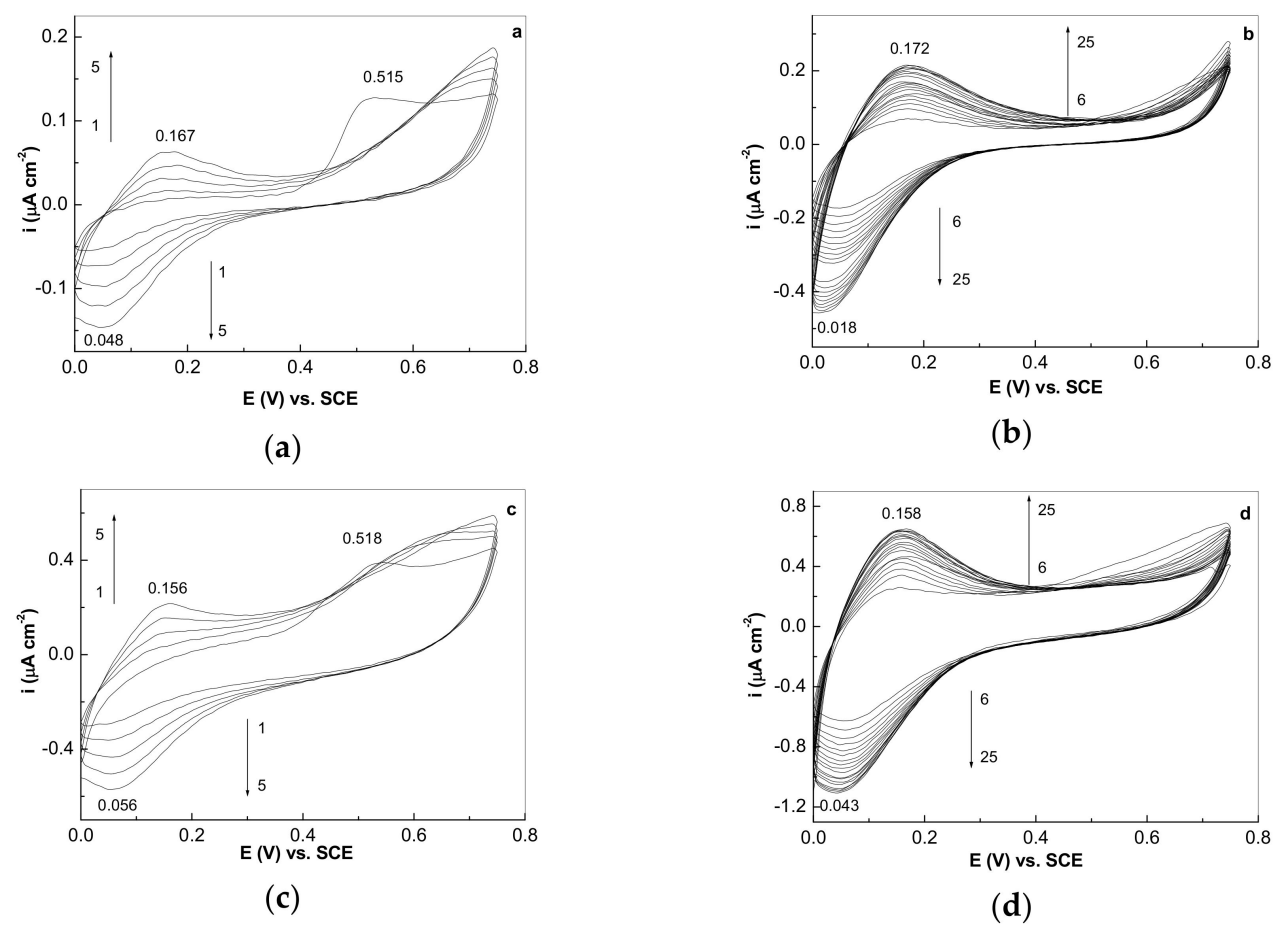
The first 1–5 and 6–25 cyclic voltammograms recorded during the electrochemical polymerization of 5 × 10^−3^ M 5A1N in the presence of 0.1 M HClO_4_ and 10^−3^ M H_4_SiW_12_O_40_ when the working electrode is the blank Au electrode (**a**,**b**) and the Au electrode is covered with the RGO sheets (**c**,**d**). The scan rate is equal to 50 mV s^−1^.

Figure 5 shows the cyclic voltammograms recorded during the electrochemical polymerization of 5A1N onto the blank Au electrode and the Au plate covered with the RGO sheets. According to Figure 5a, one observes that the cyclic voltammograms are characterized in the first cycle by an anodic peak having the oxidation potential at +515 mV and a cathodic peak having the reduction potential at +48 mV. The anodic peak with the potential at +515 mV is assigned to the 5A1N^+^ cation radical. The increasing of the number of cyclic voltammograms leads to the appearance of an anodic peak with the potential at +167 mV, which is attributed to the formation of the 5A1N^2+^ dication. During increasing the cyclic voltammogram number from 2 to 25, we note an increase in the current densities of the anodic and cathodic peaks, and simultaneously, a shift of the oxidation and reduction potentials from +167 mV and +48 mV to +172 mV and +18 mV, respectively (Figure 5a,b). In the case of the Au electrode coated with the RGO sheets, the potential of the anodic peak in the case of the first cycle is at +518 mV, this being accompanied of a cathodic peak having the potential at +56 mV. During increasing of the cyclic voltammogram number from 2 to 25, a similar behavior is remarked in Figure 5c,d to that reported in Figure 5a,b; i.e., an increase in the current densities of the anodic and cathodic peaks simultaneously with a shift of the reduction potential from +56 mV to +43 mV, respectively.

For cycles 2 to 25, the anodic peak is characterized by a potential localized between +156 and +158 mV. The increase in the current densities with increasing of the cyclic voltammograms number in Figure 5 indicates the generation of increasing P5A1N weight on the surfaces of the two working electrodes. Both in the case of the Au electrode and the Au plate coated with the RGO sheets, one observes in Figure 5b,d that the difference between the potentials of the anodic and cathodic peaks (ΔE_p_ = E_pa_ − E_pc_) is different from 57.5 mV/n (n being number of electrons), the ΔE_p_ values in the case of the 25th cyclic voltammogram being equal to 154 mV and 115 mV. As remarked on in Figure 5, regardless of the cyclic voltammogram number, the ratio between currents densities of the cathodic and anodic peaks (i_pa_/i_pc_) is not equal to unity. All these changes indicate that the irreversible processes take place at the electrode–electrolyte interface.

**Figure 6:**
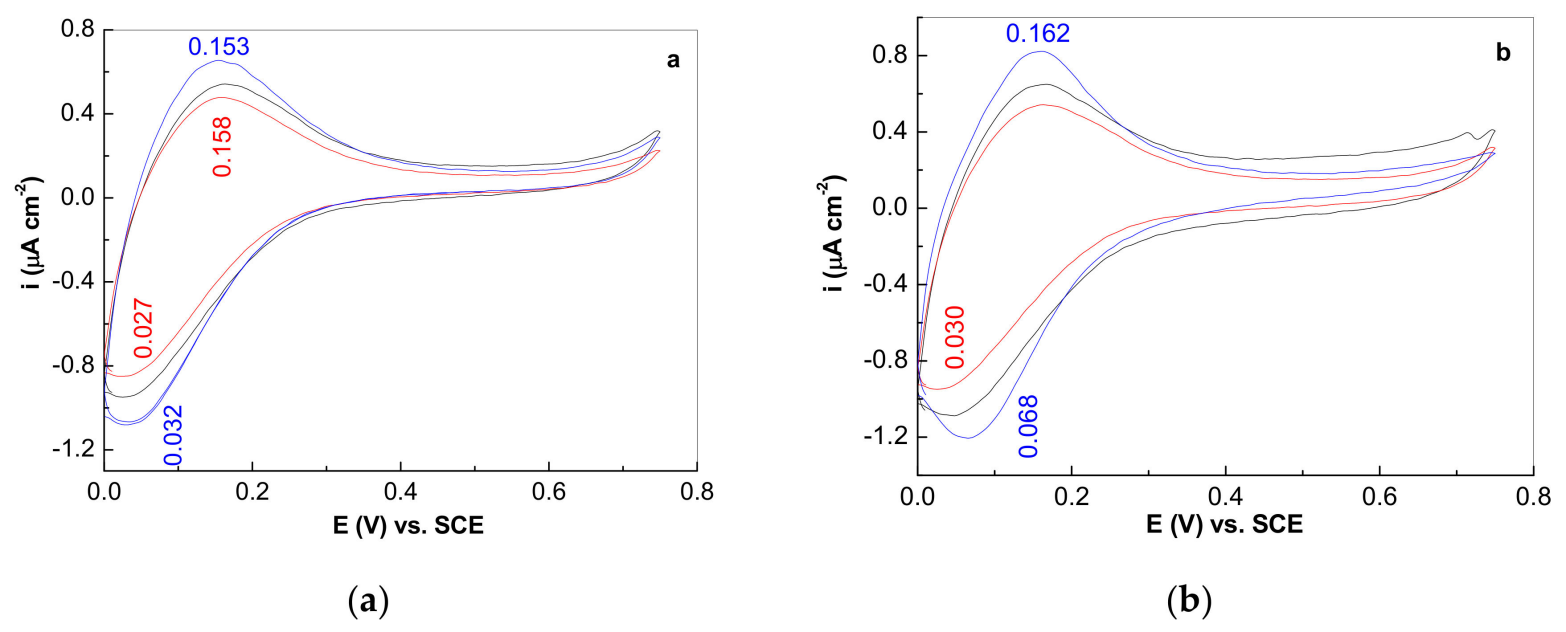
The 25th cyclic voltammogram recorded on the Au electrode (**a**) and the Au electrode covered with the RGO sheets (**b**), when the synthesis solution consists of 0.1 M HClO_4_, 10^−3^ M H_4_SiW_12_O_40_ and 5A1N with the concentration 10^−3^ M (red curve), 5 × 10^−3^ M (black curve) or 10^−2^ M (blue curve), the scan rate being equal to 50 mV s^−1^.

According to Figure 6, both in the case of the blank Au electrode and the Au electrode covered with the RGO sheets, one observes that the increasing of the 5A1N concentration induces an increase in the current densities of the cyclic voltammograms. This fact indicates a dependence of the electrochemical polymerization reaction rate with the 5A1N concentration.

**Figure 7:**
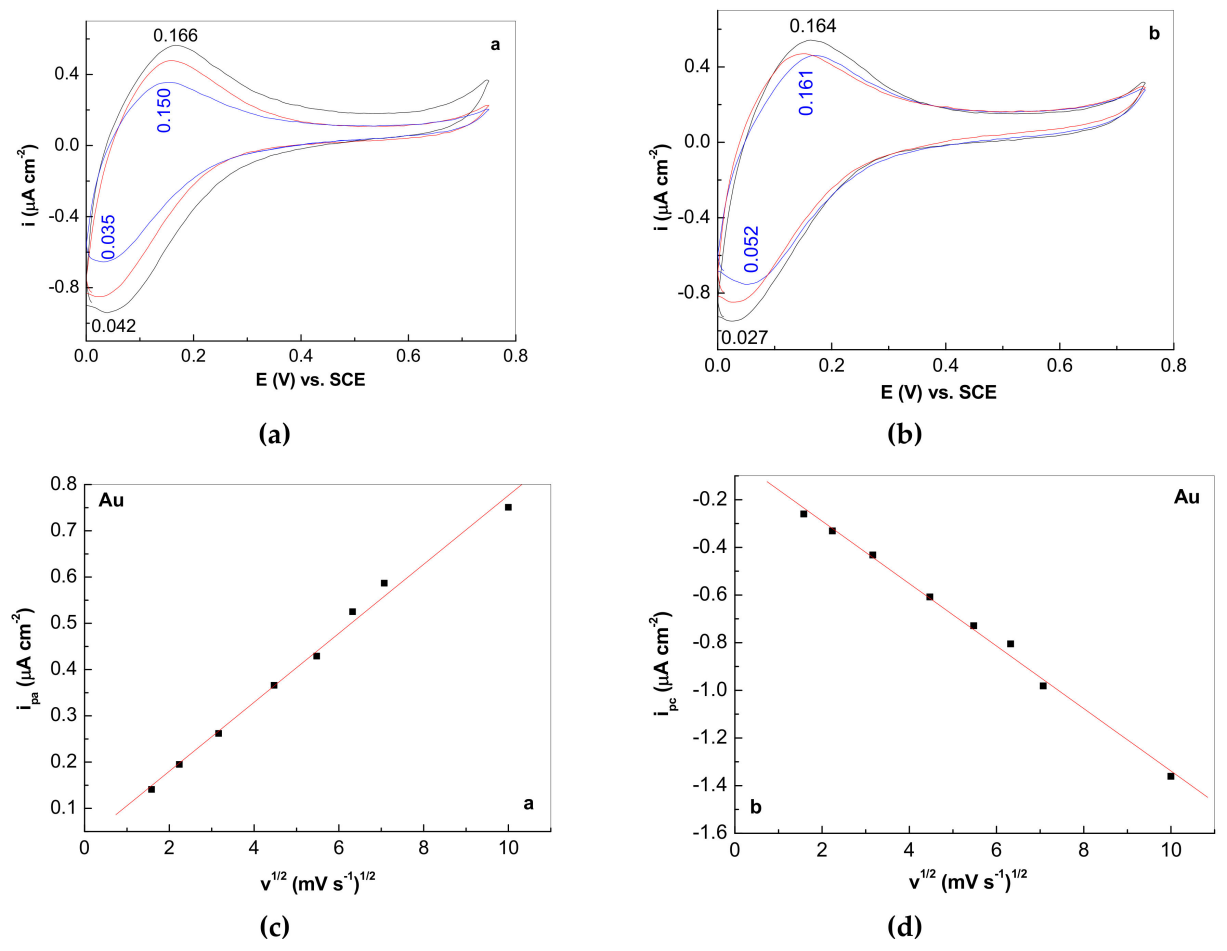

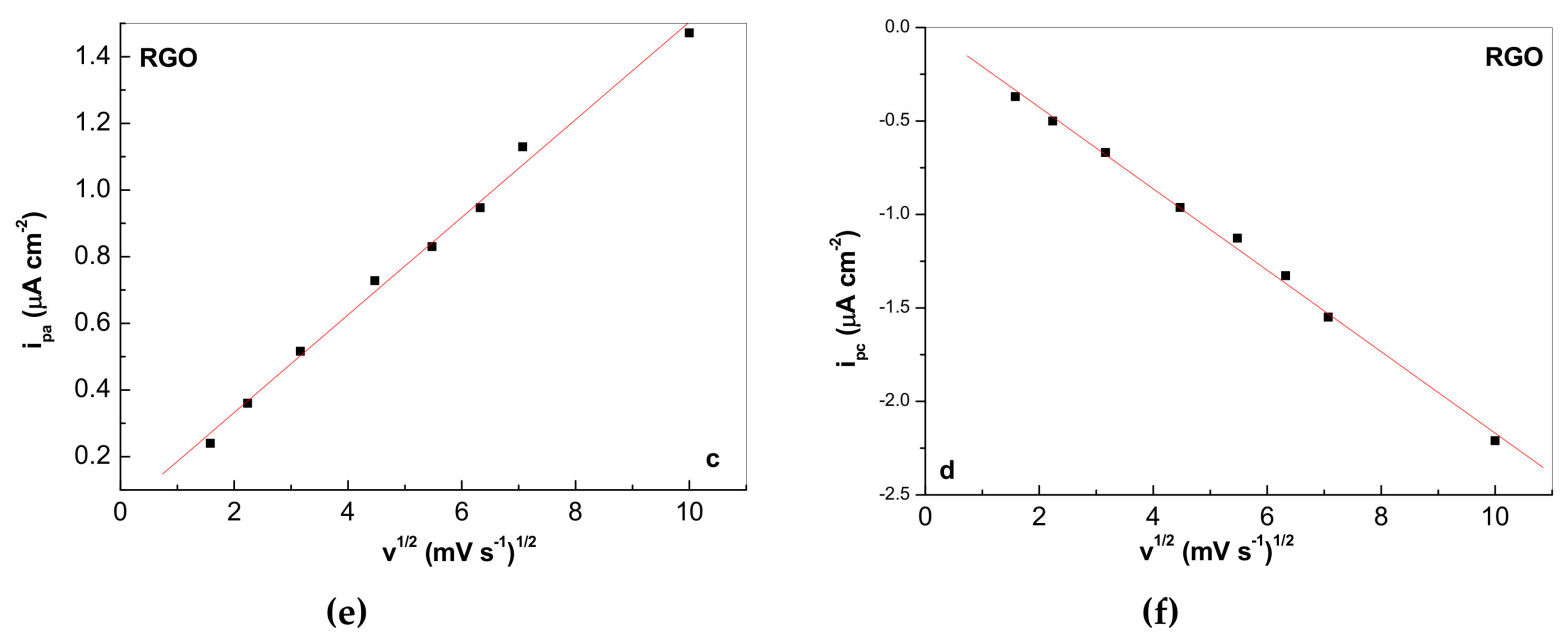
The 25th cyclic voltammogram recorded onto the blank Au electrode (**a**) and the Au electrode covered with the RGO sheets (**b**), when the synthesis solution consists of 0.1 M HClO_4_, 5 × 10^−3^ M 5A1N and H_4_SiW_12_O_40_ with the concentration 10^−3^ M (blue curve), 2 × 10^−3^ M (red curve) or 4 × 10^−3^ M (black curve), the scan rate being equal to 50 mV s^−1^, The dependence of the current densities of the anodic and cathodic peaks with the scan rate used during the 5th cyclic voltammogram recorded onto the blank Au electrode (**c**,**d**) and the Au electrode covered with the RGO sheets (**e**,**f**), when the synthesis solution consists of 10^−2^ M 5A1N, 0.1 M HClO_4_ and 2 × 10^−3^ M H_4_SiW_12_O_40_.

Regardless of the working electrode type, i.e., both in the case of the blank Au electrode and the Au electrode covered with the RGO sheets, one observes that the increasing of the H_4_SiW_12_O_40_ concentration induces an increase in the current densities of the cyclic voltammograms (Figure 7a,b). This result indicates a dependence of the 5A1N electrochemical polymerization reaction rate on the electrolyte concentration. According to Figure 7c–f, a linear dependence of the current densities of the anodic and cathodic peaks with the scan rate of the potential range (0; 0.8) V vs. SCE used for the electropolymerization of 5A1N both in the case of the blank Au electrode and the Au electrode covered with the RGO sheets is noted. This fact is the proof for the electron transfer which is controlled by diffusion. 

**Figure 8:**
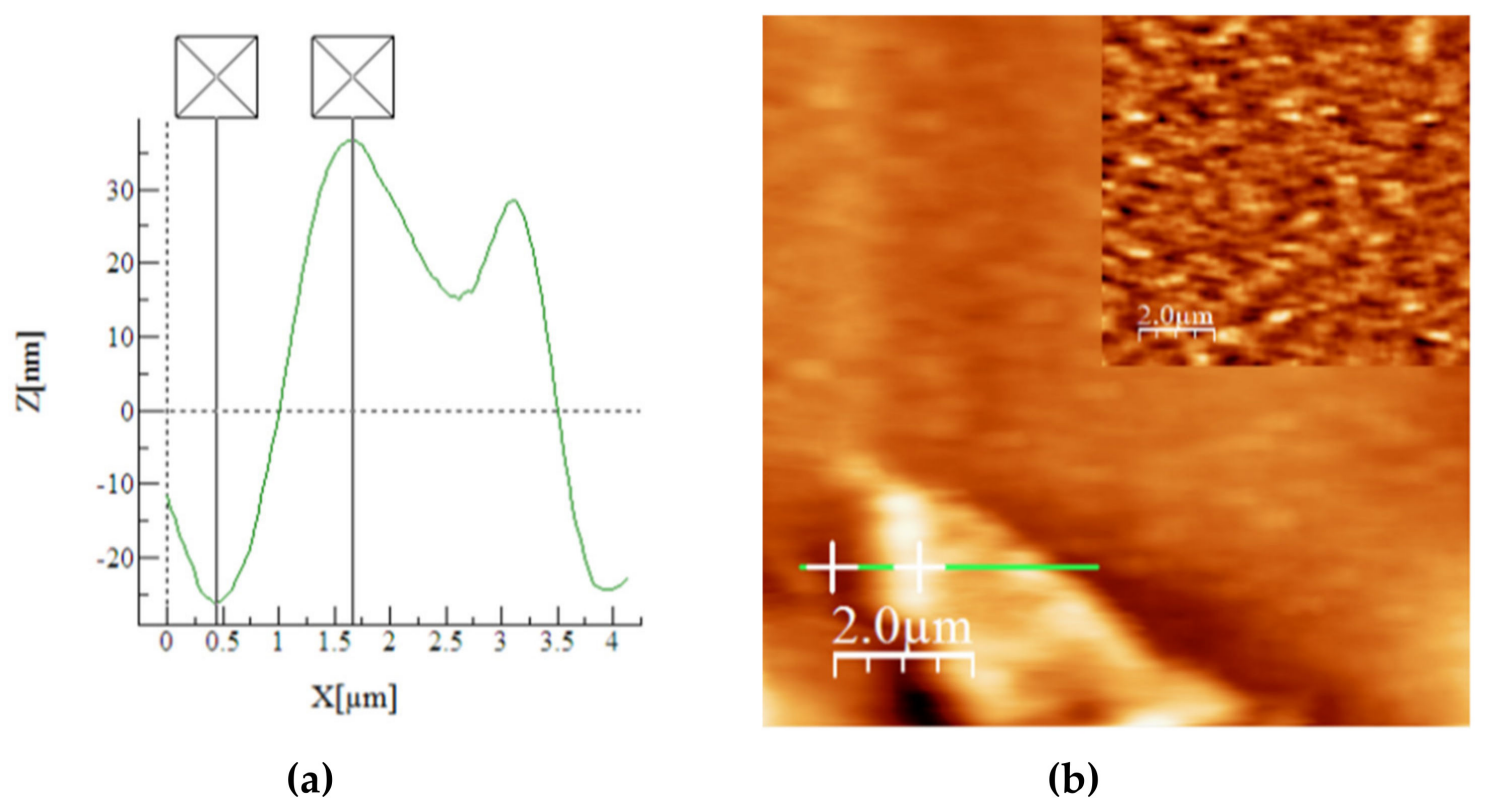
The height of the RGO sheet on the Au electrode (**a**) and atomic force microscopy (AFM) images of the blank Au electrode (insert in b) and Au electrode covered with the RGO sheets (**b**).

Additional evidences for the generation of P5A1N onto the blank Au electrode and the Au electrode covered with the RGO sheets in the following are shown by IR spectroscopy and Raman scattering. In this stage of our studies, a short description of the roughness parameters of the Au electrode before and after coating with the RGO sheets is necessary. According to Figure 8, the Au layer is very smooth, being characterised by low roughness parameters: 1.5 nm RMS and 1.1 nm Ra. The deposition of the RGO sheets onto the Au electrode surface induces an increase in roughness parameters at 17 nm RMS and 13 nm Ra. The height of the RGO sheet calculated from AFM was of 60 nm. 

**Figure 9:**
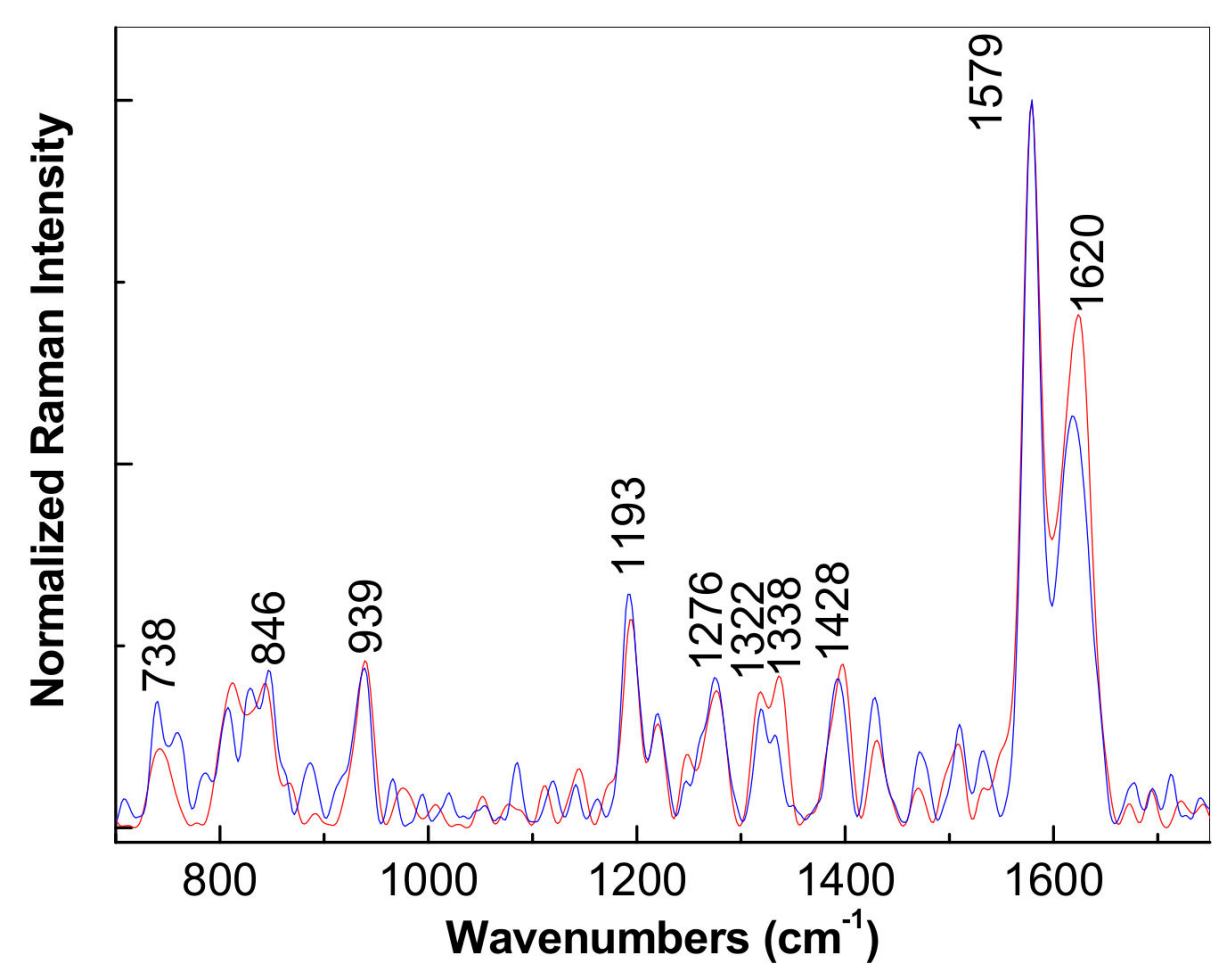
The Raman spectrum of P5A1N electrosynthetized onto the blank Au electrode (blue curve) and the Au electrode covered with the RGO sheets (red curve).

According to Figure 9, the main Raman lines of P5A1N electrosynthesized onto the Au electrode have peaks at 738–846–939, 1193, 1276, 1322–1338, 1428, 1579 and 1620 cm^–1^, these being assigned to the vibrational modes of the C–H bond, the deformation of the C–C bond in naphthalene nuclei, stretching of the C–H bond, stretching of the C–C and C–O bonds, bending of the C–N bonds and the stretching of the C–C and C=C bonds in naphthalene nuclei [10,11].

The Raman spectrum of P5A1N electrosynthesized onto the Au electrode covered with the RGO sheets highlights as the main difference in comparison to the P5A1N electrogenerated onto the blank Au electrode, a change in the value between the intensities of the Raman lines peaking at 1579 and 1620 cm^−1^ from 1.46 (blue curve in Figure 9) to 1.87 (red curve in Figure 9). This fact can be explained as a consequence of the superposition of the Raman lines of P5A1N with those of RGO in the spectral range 1550–1650 cm^−1^ [13,14]. 

**Figure 10:**
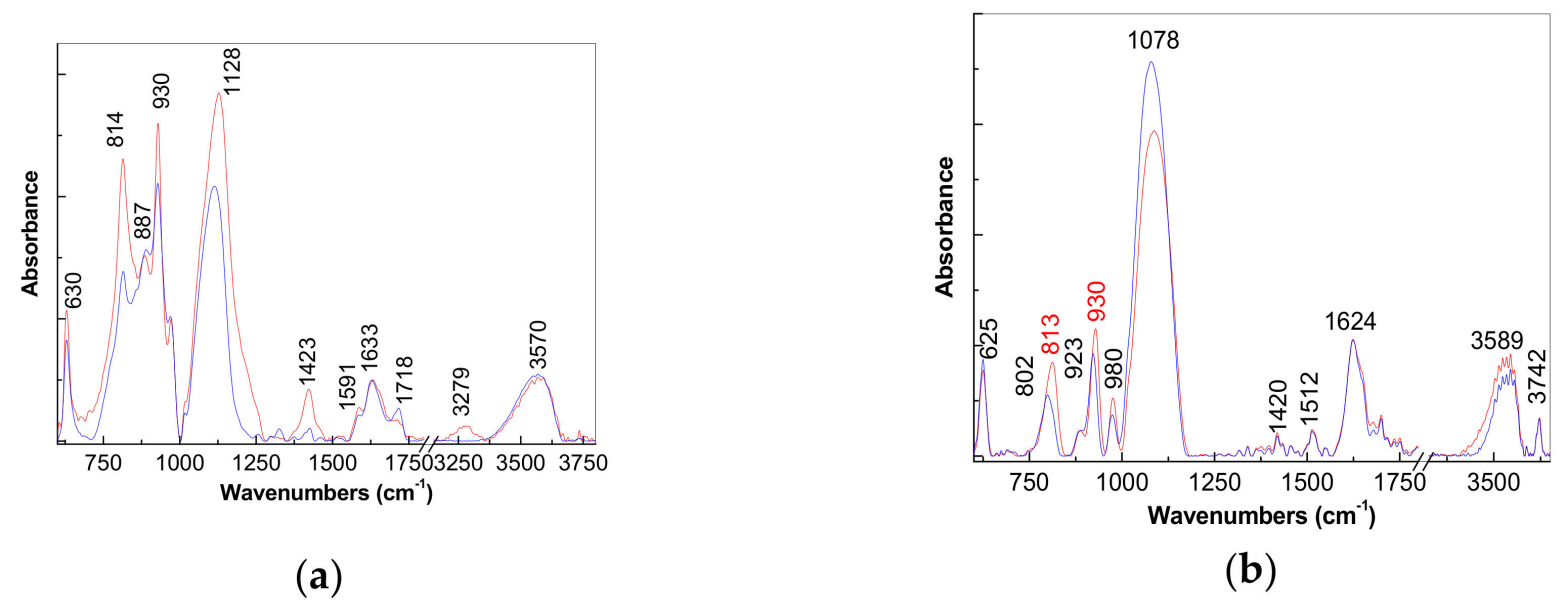
The IR spectra of P5A1N electrochemical synthetized onto the blank Au electrode (**a**) and the Au electrode covered with the RGO sheets (**b**) by recording 5 (blue curve) and 25 (red curve) cyclic voltammograms.

**Scheme 2:**
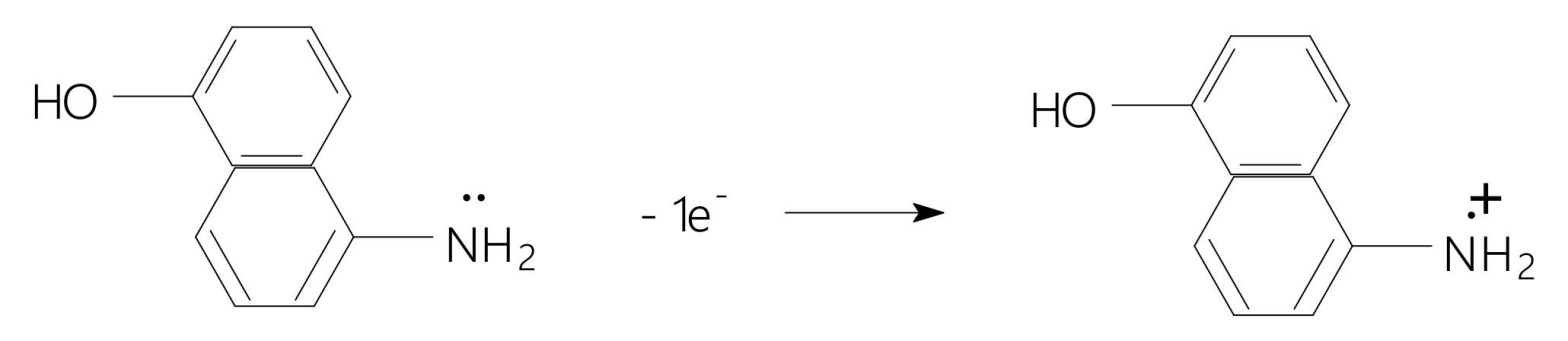

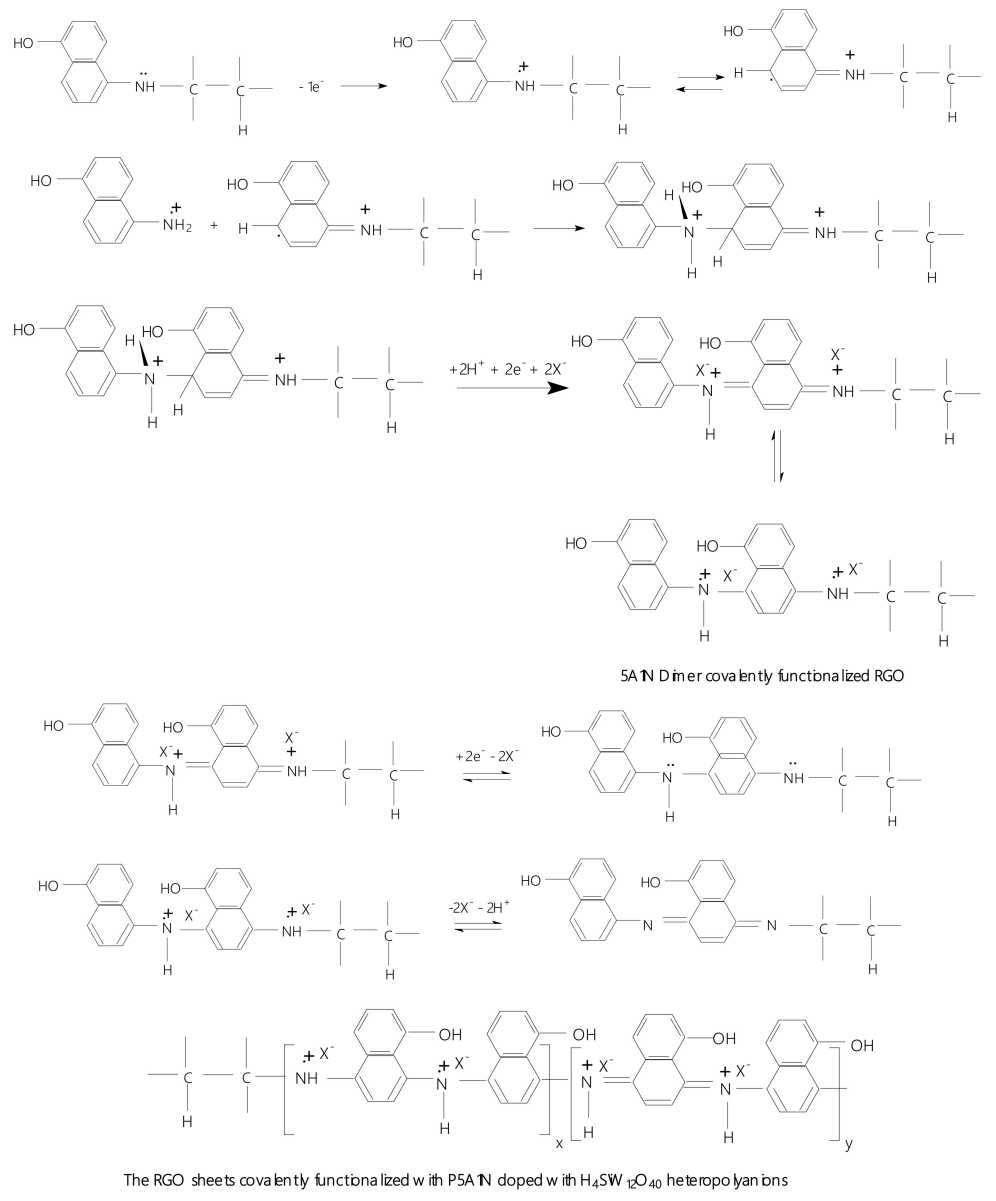
The mechanism of the electrochemical polymerization of 5A1N in the presence of the RGO sheets, X^−^ corresponds to the H_4_SiW_12_O_40_ heteropolyanions; i.e., H_3_SiW_12_O_40_^−^. In this scheme, x and y correspond to the numbers of the reduced and oxidized entities of repeating units of macromolecular compound.

Other vibrational changes in the case of P5A1N electrosynthetized onto the blank Au electrode and the Au electrode covered with the RGO sheets are reported in the following by IR spectroscopy. In this context, Figure 10a highlights the main IR bands of the P5A1N synthetized in the presence of HClO_4_ and H_4_SiW_12_O_40_ on the Au electrode, which have peaks at 630, 814, 1128, 1423, 1591, 1633 and 3570 cm^−1^, these being assigned to the vibrational modes of the deformation of the naphthalene ring, out-of-plane bending of the C–H bond of naphthalene nuclei, in-plane bending of the C–H bonds of naphthalene nuclei, stretching of the C–O bond of phenolic compounds overlapping the in-plane bending of the O–H bond, stretching of the C=C and C–H bonds of naphthalene, stretching of the C=N bonds and stretching of the N–H bond in functional groups R–NH_2_ [1]. The IR bands at 887–930 and 1718 cm^−1^ are attributed to the vibrational mode of the W–O–W bond (octahedral corner-sharing) of the H_4_SiW_12_O_40_ heteropolyanions and the stretching of the O–H bond in water coming from H_4_SiW_12_O_40_ xH_2_O [1,23]. Increasing the cyclic voltammograms number recorded onto the Au electrode induces an increase in the absorbance of the IR bands situated at 630, 814, 930, 1128 and 1423 cm^−1^. The main differences in the case of the electrosynthesis of P5A1N onto the Au electrode covered with the RGO sheets (Figure 10b) in contrast with the Au electrode (Figure 10a) are: (i) a decrease in the absorbance of the IR bands situated in the spectral range 850–1000 cm^−1^ simultaneously with the appearance of the IR band with maximum at 980 cm^-1^ which belongs to the vibrational mode W-O of the H_4_SiW_12_O_40_ heteropolyanions; (ii) a down-shift of the IR bands from 630, 1128 and 1633 cm^−1^ to 625, 1078 and 1624 cm^−1^, accompanied by a change of the ratio between the absorbance of the IR bands at 813–930 and 1000–1200 cm^−1^; and iii) an up-shift of the IR band from 3570 to 3589 cm^−1^ simultaneously with the appearance of a new IR band at 3742 cm^−1^. The new IR band at 3742 cm^−1^ belongs to the stretching vibrational mode of the N-H bond in the R-NH-R functional groups [19]. The presence of this additional bond in the case of P5A1N electrosynthetized onto the RGO sheets can only be explained by considering the mechanism of the electrochemical polymerization shown in Scheme 2. According to Scheme 2, the reaction product of the electrochemical polymerization of 5A1N on the Au electrode covered with the RGO sheets consists of the RGO sheets covalently functionalized with P5A1N doped with the H_4_SiW_12_O_40_ heteropolyanions. In our opinion, Scheme 2 explains: (i) the presence of the R-NH-R functional group, as a consequence of the covalent functionalization of the RGO sheets with P5A1N, evidenced by the IR band at 3742 cm^−1^; and (ii) the down-shift of the IR bands assigned to the vibrational modes of the deformation of the naphthalene ring, stretching of the C–O bond of phenolic compounds overlapping the in-plane bending of the O-H bond and stretching of the C=N bonds as a result of the covalent bonding of P5A1N in doped state onto the RGO sheets’ surface.

### 3.3. Chemical Adsorption of PDITC onto the RGO Sheets Covalently Functionalized with P5A1N

**Figure 11:**
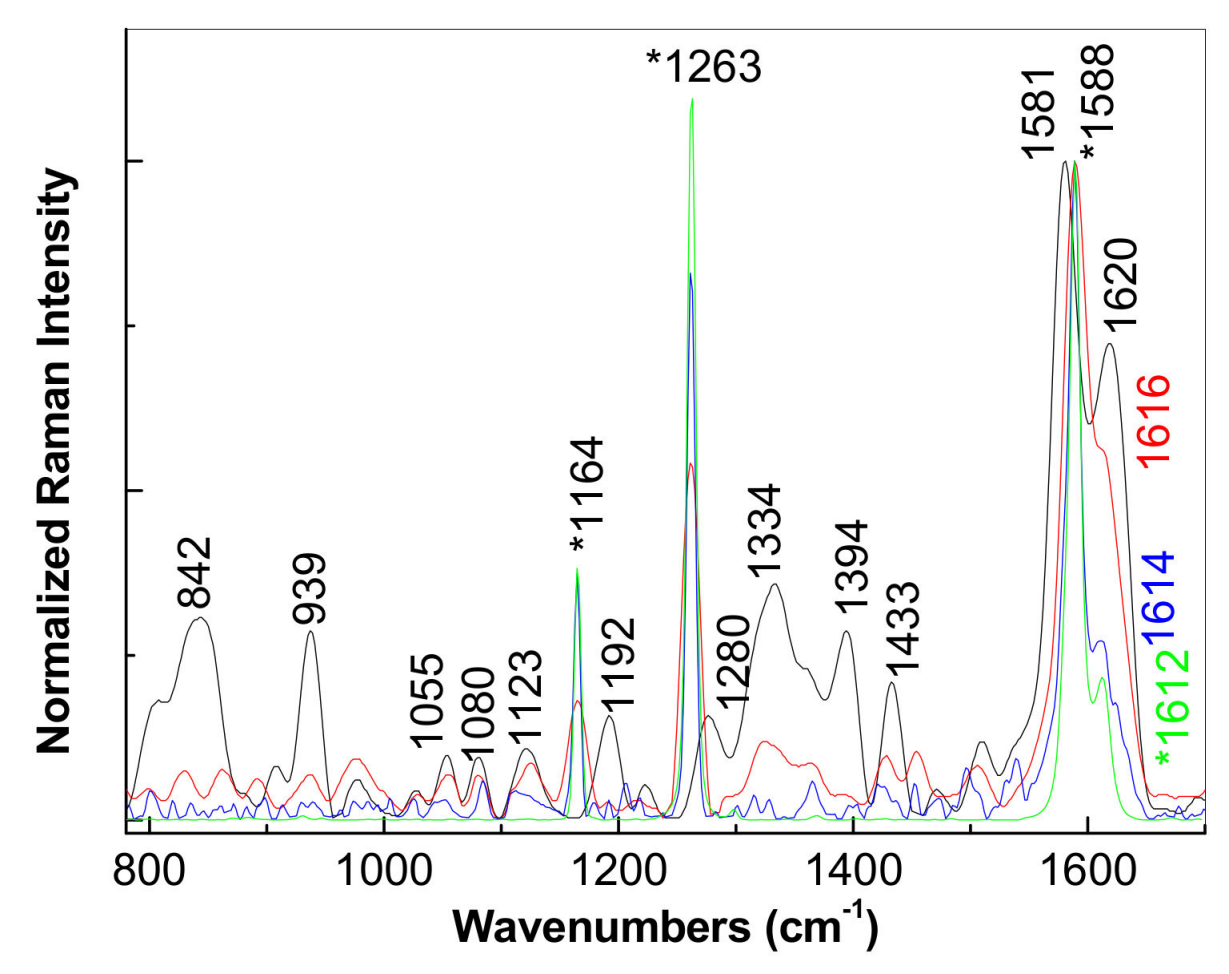
The Raman spectra of PDITC (green curve) before and after the adsorption onto the Au electrode covered with the RGO sheets covalently functionalized with P5A1N doped with H_4_SiW_12_O_40_ heteropolyanions using 2 mL (blue curve), 1 mL (red curve) and 0.5 mL (black curve) of the PDITC in C_2_H_5_OH solution at the concentration of 1 mg/mL.

Figure 11 shows the Raman spectra of PDITC before and after the interaction with the RGO sheets covalently functionalized with P5A1N doped with H_4_SiW_12_O_40_ heteropolyanions.

As observed in Figure 11, the Raman spectrum of PDITC deposited onto the Au electrode shows lines situated at 1164, 1263, 1588 and 1612 cm^−1^, these being assigned to the vibrational modes: C–S bending, C–H in benzene ring + C–C stretching + C–N stretching, C=C + C–C stretching in benzene ring and C–C stretching + C–H bending in benzene ring, respectively [24,25,26]. The interaction of PDITC with the RGO sheets covalently functionalized with P5A1N doped with H_4_SiW_12_O_40_ heteropolyanions induces the following changes in the Raman spectra of the two constituents: (i) a gradual decrease in the intensity of the Raman lines situated in the spectral range and 1300–1500 cm^−1^; (ii) the disappearance of the Raman peaks of 842, 939 and 1192 cm^−1^; (iii) an intense Raman line with a maximum at 1588 cm^−1^ is observed regardless of the PDITC weight interacted with the RGO sheets covalently functionalized with P5A1N doped with H_4_SiW_12_O_40_ heteropolyanions; (iv) a gradual down-shift of the Raman line from 1620 to 1614 cm^−1^ while increasing the PDITC weight that has interacted with the RGO sheets covalently functionalized with P5A1N doped with H_4_SiW_12_O_40_ heteropolyanions; (v) a change of the ratio between the intensities of the Raman lines at 1588 and 1612–1620 cm^−1^ from 4 (green curve in Figure 9) to 1.5 (black curve in Figure 9); and (vi) the appearance of new Raman lines at 1055–1080–1123 cm^−1^. 

**Figure 12:**
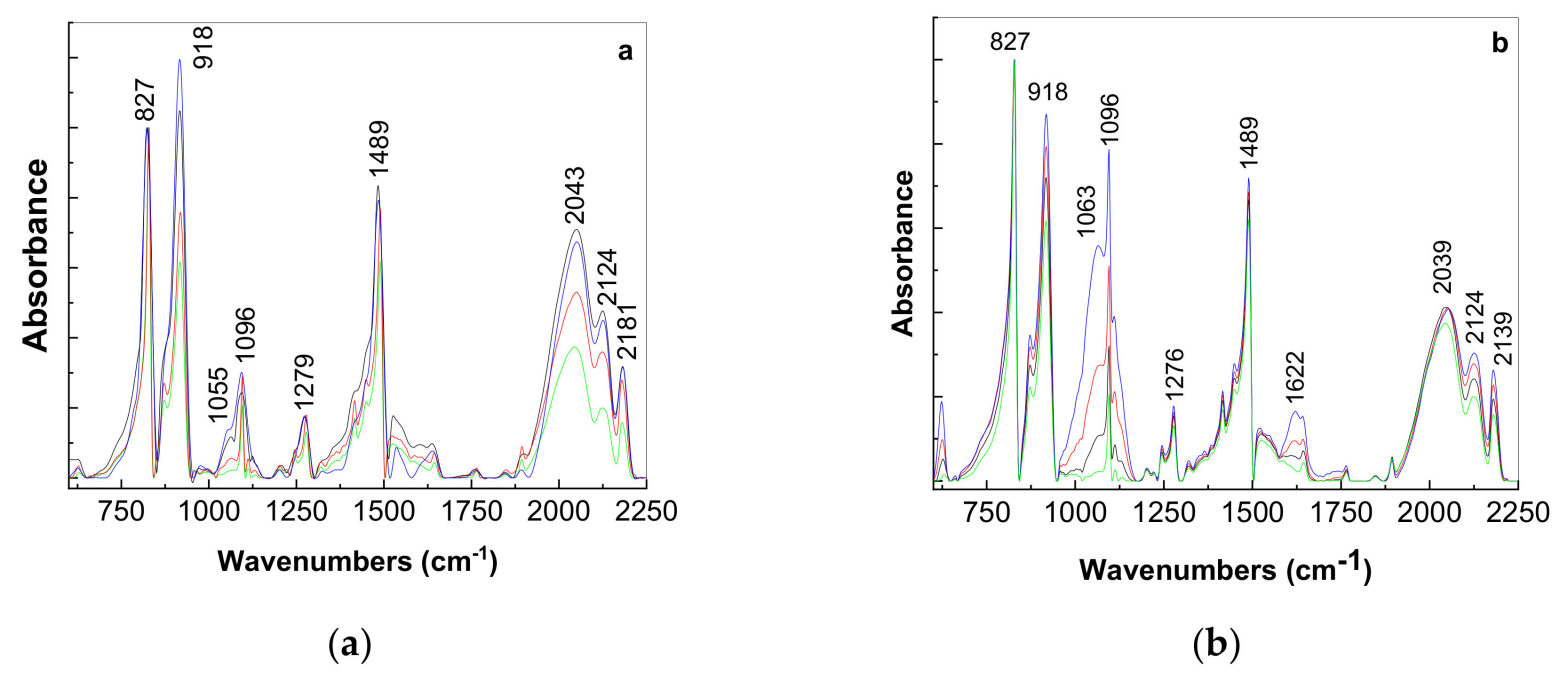
The IR spectra of PDITC (green curve) before and after the adsorption onto the blank Au electrode (**a**) and the Au electrode covered with the RGO sheets covalently functionalized with P5A1N doped with H_4_SiW_12_O_40_ heteropolyanions (**b**) using 2 mL (red curve), 1 mL (black curve) and 0.5 mL (blue curve) of the PDITC in C_2_H_5_OH solution with the concentration of 1 mg/mL.

**Scheme 3:**
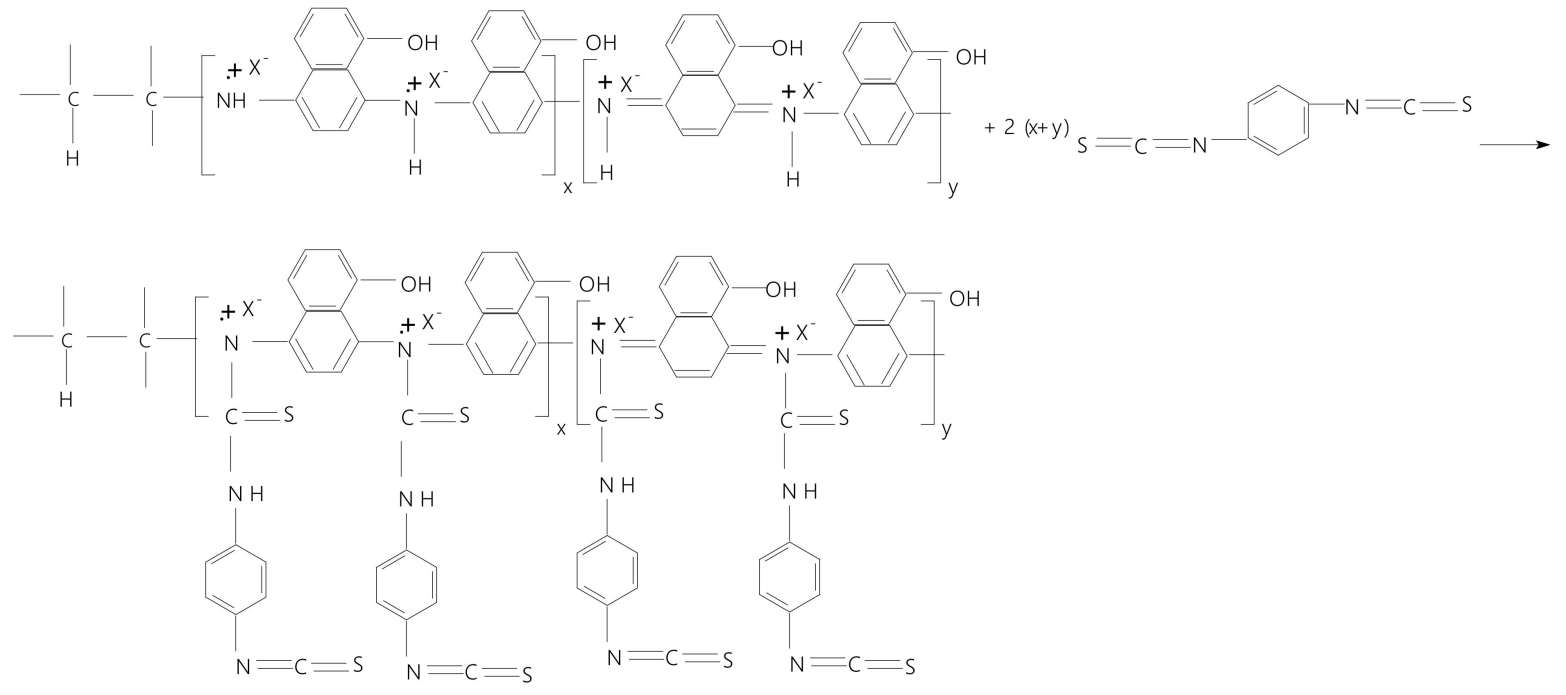
The chemical interaction of PDITC onto the RGO sheets covalently functionalized with P5A1N doped with H_4_SiW_12_O_40_ heteropolyanions. In this scheme, x and y correspond to the number of the reduced and oxidized entities of repeating units of macromolecular compound.

According to Figure 12, the main IR bands of PDITC are localized at 827, 918, 1055–1096, 1279– 1489 and 2043–2124–2181 cm^–1^, these being assigned to the vibrational modes of bending of C–H out-of-plane in benzene, p–substituted; C–H bending; C=S; C=N in thiourea; and N=C=S [27,28]. The interaction of PDITC with P5A1N doped with H_4_SiW_12_O_40_ heteropolyanions electrosynthesized onto the Au electrode induces increases in the absorbances of the IR bands at 918, 1055 and 2043–2124–2181 cm^–1^ (Figure 12a). Significant differences in the case of the PDITC interacting with the P5A1N doped with H_4_SiW_12_O_40_ heteropolyanions electrosynthesized onto the Au electrode covered with the RGO sheets were observed concerning the up-shift of the IR band from 1055 to 1063 cm^–1^ and the change of the ratio between the absorbance of IR bands at 1096 and 918/827/1489 cm^–1^ (A_1096_/A_918;_ A_1096_/A_827;_ A_1096_/A_1489_) (Figure 12b). The increase in the absorbance of the IR band at 1096 cm^–1^ indicates the presence of some hindrance steric effects induced as a consequence of the fact that the adsorption PDITC onto the RGO sheets covalently functionalized with P5A1N doped with H_4_SiW_12_O_40_ heteropolyanions involves a chemical interaction as shown in Scheme 3.

In our opinion, Scheme 3 explains: (i) the variation of the ratio between the intensities of the Raman lines at 1588 and 1612–1620 cm^−1^ from 4 to 1.5 in Figure 11; (ii) the new Raman lines at 1055–1080–1123 cm^−1^, which were reported in substituted thiourea; and (iii) the enhancement in the absorbance of the IR band at 1096 cm^−1^ (Figure 12) which was assigned to the C=S vibrational mode of the substituted thiourea.

## 4. Conclusions

In this work, new results were reported concerning the electrochemical polymerization of 5A1N in the presence of HClO_4_, H_4_SiW_12_O_40_ and the RGO sheets by cyclic voltammetry, Raman scattering and IR spectroscopy. These results have highlighted that: (i) using Raman scattering and IR spectroscopy a chemical interaction between 5A1N and the RGO sheets has been demonstrated; (ii) the electrochemical polymerization reaction of 5A1N in the presence of HClO_4_ and H_4_SiW_12_O_40_ when as working electrode is used an Au plate covered with the RGO layers leads to the RGO sheets covalently functionalized with P5A1N doped with the H_4_SiW_12_O_40_ heteropolyanions according to the Raman scattering and IR spectroscopy studies; in this context, we have proposed a mechanism for the electrosynthesis of the RGO sheets covalently functionalized with P5A1N doped with the H_4_SiW_12_O_40_ heteropolyanions; and (iii) the adsorption of PDITC onto the RGO sheets covalently functionalized with P5A1N doped with the H_4_SiW_12_O_40_ heteropolyanions involves a chemical reaction which induces the appearance of the functional groups similar to the substituted thiourea.
